# Increased Risk of Obesity Resulting from the Interaction between High Energy Intake and the Trp64Arg Polymorphism of the *β*_3_-adrenergic Receptor Gene in Healthy Japanese Men

**DOI:** 10.2188/jea.15.203

**Published:** 2005-11-07

**Authors:** Koichi Miyaki, Shinya Sutani, Haruhito Kikuchi, Izumi Takei, Mitsuru Murata, Kiyoaki Watanabe, Kazuyuki Omae

**Affiliations:** 1 Department of Preventive Medicine and Public Health, School of Medicine, Keio University.; 2 Department of Laboratory Medicine, School of Medicine, Keio University.; 3 Department of Internal Medicine, School of Medicine, Keio University.

**Keywords:** Obesity, Receptors, Adrenergic, beta-3, Polymorphism, Single Nucleotide, Energy Intake, Questionnaires

## Abstract

BACKGROUND: Few studies have investigated the interaction between the Trp64Arg polymorphism of the *β*_3_-adrenergic receptor gene (*ADRB3*) and environmental factors. This study aimed to investigate whether energy intake affects the relationship between this polymorphism and obesity.

METHODS: Healthy Japanese men (n=295; age 46.1±11.5 years (mean ± standard deviation); waist circumference 83.9±9.3 cm; body mass index (BMI) 23.3±3.3 kg/m^2^) recruited in a Japanese chemical industry firm were eligible for analysis. Daily energy intake, protein, fat, and carbohydrate (PFC) ratio and daily physical activity were assessed by self-reported questionnaires. Genotyping for the polymorphism was performed with written informed consent.

RESULTS: When the subjects were classified into two groups according to presence of the polymorphism, the groups were not significantly different in waist circumference or BMI. Quartile classification of energy intake, however, demonstrated a significantly larger ratio of obese subjects to non-obese subjects in the group with the polymorphism in the highest 4th quartile alone. Multiple logistic regression analysis also revealed that the presence of the polymorphism increased the risk of obesity significantly in the 4th quartile alone (adjusted odds ratio=3.37, 95% confidence interval=1.12-10.2).

CONCLUSION: Presence of the polymorphism alone does not significantly increase the risk of obesity. However, high energy intake interacts with the polymorphism and leads to a significant increase in risk of obesity. The Trp64Arg polymorphism of *ADRB3* warrants consideration, along with other polymorphisms involved in the development of obesity, for tailor-made prevention of obesity.

Obesity is attributable to the effects of genetic and environmental factors, and it affects human health.^1^ Accumulation of visceral fat, in particular, may play an important role in the pathogenesis of glucose intolerance, hyperlipidemia, and hypertension, and the accumulation of visceral fat is closely linked with atherosclerosis.^2^^-^^4^

Many genes are suspected to be associated with obesity,^5^ and recently the *β*_3_-adrenergic receptor gene (*ADRB3*) has become the center of attention. In humans *ADRB3* is predominantly expressed in visceral adipose tissue.^6^ It contains seven transmembrane domains and is coupled with G proteins.^7^ Stimulation of the *ADRB3* by *β*-adrenergic agonists has been demonstrated to activate adenylate cyclase, which increases the intracellular cyclic AMP level and leads to acceleration of lipid metabolism and ther-mogenesis.^7^

In 1995, Pima Indians, an ethnic group with a very high prevalence of obesity and non-insulin-dependent diabetes mellitus (NIDDM), were found to have a high frequency of the Trp64Arg polymorphism due to the replacement of thymidine (T) by cytosine (C) at nucleotide position 190 in *ADRB3*, and those with the polymorphism were found to have early onset of NIDDM and a low resting metabolic rate.^8^ Subjects with the polymorphism in France have been found to have increased capacity to gain weight,^9^ and the polymorphism is associated with abdominal obesity in Finns and may cause insulin resistance and earlier onset of NIDDM in that population.^10^

The polymorphism has been detected at an allelic frequency of about 0.20 in Japanese, which is lower than in Pima Indians (0.31) and higher than in Mexican-Americans (0.13), Blacks (0.12) and Whites (0.08) in the United States.^8^^,^^11^ The resting metabolic rate of obese Japanese women with the polymorphism is about 200 kcal/day lower than of those without the polymorphism, and women with the polymorphism have difficulty in losing weight.^12^ Moreover, the polymorphism has been found to be associated with visceral-fat obesity and insulin resistance syndrome, and it may be possible to use the polymorphism as a genetic marker for these syndromes.^13^ Other studies have also shown that the polymorphism is associated with obesity^14^^,^^15^ and insulin resistance.^15^

A meta-analysis of the association between the polymorphism of *ADRB3* and body mass index (BMI) in 31 studies demonstrated that subjects with the polymorphism had a BMI that averaged 0.30 (95% confidence interval [CI]=0.13-0.47) higher than those without the polymorphism.^16^

Many studies have demonstrated that the polymorphism of *ADRB3* is associated with obesity, but few studies have shown that it is possible to prevent or treat obesity and its complications in persons with the polymorphism. Therefore, to achieve tailor-made prevention or treatment based on genotype, including *ADRB3*, we investigated the interaction between energy intake and the polymorphism in *ADRB3* in healthy Japanese men.

## METHODS

This study was approved by the Institutional Review Board (IRB) of Keio University School of Medicine, and was carried out in concordance with the principles of the Declaration of Helsinki. We explained this study by means of a written document to healthy Japanese men and women working for a company in Kanagawa prefecture and obtained written informed consent from 363 workers (male: 326, female: 37) to participate in the study, which included genotyping. The following surveys and genotyping for the polymorphism of ADRB3 were conducted in October and November 2003, and we obtained complete replies to the food frequency questionnaire (FFQ) from 337 (male: 295, female: 32) of the 363 workers, who were genotyped for the polymorphism of *ADRB3*. Allowing for the fact that the Japan Society for the Study of Obesity established different criteria for ‘obesity disease’ in men and women,^17^ and the report that men might have a higher risk of coronary heart disease than women due to the difference in body fat distribution,^18^ we performed the analysis focused on the 295 male workers.

Their mean age, waist circumference, and BMI (mean ± standard deviation) were 46.1±11.5 years, 83.9±9.3 cm, and 23.3±3.3 kg/m^2^, respectively. Height, weight, waist circumference, systolic and diastolic blood pressure, fasting plasma glucose level, and serum lipid levels were measured in all the subjects. A Body mass index (BMI) was calculated as weight (kg)/height^2^ (m^2^). Obesity was defined as BMI 25+ kg/m^2^ or waist circumference 85+ cm, based on the criteria for ‘obesity’ of the Japan Society for the Study of Obesity.^17^ In addition, information on the age, physical activity, smoking status, energy intake, and the protein, fat, and carbohydrate (PFC) ratio of all the subjects was obtained by means of a self-report questionnaire. The semi-quantitative food frequency questionnaire (FFQ), which was evaluated by comparing the FFQ with the 7-day dietary records of 66 subjects by Takahasi K, et al.^19^, was used to calculate energy intake and PFC ratio. Takahasi K, et al. reported that the correlation coefficients between the FFQ and the 7-day dietary records for energy, protein, fat, and carbohydrate intake were 0.47 (p<0.001), 0.42 (p<0.001), 0.39 (p<0.01), and 0.49 (p<0.001), respectively.^19^

Daily physical activity was calculated based on hours per day subjects engaged in each activity. The intensity of each activity was quantified by assigning metabolic equivalents (METs) to each activity.^20^ METs represent the ratio of energy expended during each specific activity to resting metabolic rate. The time spent in each activity was multiplied by the specific number of METs assigned to each activity, and the products were summed. The sum was defined as the daily physical activity (METs • minutes) of each subject.

The FFQ used in this study asked about the consumption of food items in 29 food groups during the previous one or two months. Basically, the subjects were asked to describe the quantities and frequencies of consumption of food items during breakfast, lunch, and dinner. Three portion size categories (small, medium, and large) were used to evaluate the quantities of consumption of food items. “Small” is half the size of “medium”, and “large” is one and a half times the size of “medium”. When the frequency of consumption of a food is less than once or twice a month, the subject is instructed to answer “never”. The FFQ illustrated the food items of each food group in quantity equal to “medium”, so as to evaluate the quantities of consumption correctly. When the frequency of consumption of a food was low, the subjects were asked to answer the quantity of consumed at one time and the frequency of consumption per week. Foods of this kind included sea vegetables, fruit, potatoes, butter, pickles, etc. The subjects were also asked to describe the quantity of consumption at one time and the frequency of consumption per week of alcoholic and non-alcoholic beverages. When the quantity of a unit of a food was almost identical among subjects, the subjects were asked to state only the frequency of consumption per week as the number of consumed units. Foods of this kind included rice, milk, egg, etc. Daily nutrient intake was calculated by multiplying the frequency of consumption of each food by the nutrient content of the portion size and summing the products for all foods items. The energy intake and PFC ratio of each subject were determined in this manner.

A peripheral blood specimen was collected from each subject, and genotyping for the polymorphism of *ADRB3* was performed by polymerase chain reaction (PCR) and single nucleotide primer extension (SNuPE) assay with Ampdirect (Shimadzu Corporation, Kyoto, Japan), which eliminates the DNA extraction process and amplifies the genomic DNA directly from the whole blood.

A 367-bp fragment of the gene encompassing the polymorphism site was amplified by PCR using 5′-primer (5′TTC-CTTCTTTCCCTACCGCCC)^8^ and 3′-primer (5′GCAGCCAGTG-GCGCCCAACGG).^8^ The PCR reactions were carried out in the PCR mixture containing 10 *μ*L of Ampdirect-G/C (Shimadzu Corporation, Kyoto, Japan), 10 *μ*L of Ampdirect Addition (Shimadzu Corporation, Kyoto, Japan), 4 *μ*L of dNTP mixture (TaKaRa Bio Inc., Shiga, Japan), 0.5 *μ*M of 5′-primer,^8^ 0.5 *μ*M of 3′-primer,^8^ 0.25 *μ*L of *Taq* DNA Polymerase (Promega Corporation, Wisconsin, USA), up to 50 *μ*L of distilled water, and 1.0 *μ*L of whole blood. The PCR consisted of preheating at 80°C for 15 minutes; denaturation at 94°C for 4.5 minutes; 40 cycles of denaturation at 94°C for 30 seconds, annealing at 60°C for 1 minute, and extension at 72°C for 1 minute; a final extension at 72°C for 7 minutes. The SNuPE assay was then performed. The SNuPE assay was based on the incorporation of a single fluorescent-labeled ddNTP, which was correctly paired with the template DNA and caused chain-termination, to the 3′ terminus of a primer annealed next to the polymorphic site.^21^ The SNuPE primer (5′ATGGTCTGGAGTCTCGGAGTCC) was designed so that the primer ends immediately before the polymorphic site. The SNuPE reactions were carried out in the mixture; containing 2 *μ*L of PCR product, 4 *μ*L of SNuPE premix (ddATP, ddCTP, ddGTP, ddTTP, DNA polymerase), and 2 pM of SNuPE primer. The SNuPE consisted of 25 cycles of denaturation at 94°C for 10 seconds, annealing at 53°C for 5 seconds, and extension at 60°C for 10 seconds. The products were analyzed with ABI 7700 (Applied Biosystems, California, USA).

For statistical analysis, Student’s t-test was used to compare normally distributed variables between groups. Variables that were not normally distributed were log transformed. When the log transformations of the variables were effective, Student’s t-test was used. When ineffective, the Wilcoxon rank-sum test was used. The *χ*^2^ test was used to compare categorical variables. Multiple logistic regression analysis was performed on obesity defined as waist circumference 85+ cm,^17^ with presence of the polymorphism of *ADRB3*, age, smoking, and physical activity as variables.

Differences were assessed by two-sided tests, with an alpha level of 0.05. All statistical analyses were performed with Statistical Package for the Social Sciences^®^ (SPSS) for Windows, version 11 software (SPSS Inc., Illinois, USA).

## RESULTS

Genotyping for the Trp64Arg polymorphism of *ADRB3* in the 295 healthy Japanese male subjects showed that 198 were homozygous for the wild-type allele (Trp/Trp), 94 were heterozygous for the variant allele (Arg/Trp), and 3 were homozygous for the variant allele (Arg/Arg) (allelic frequency=0.17). These results were in Hardy-Weinberg equilibrium and did not conflict with the results previously reported in another Japanese population (p=0.240).^13^

The main characteristics of the subjects are shown in Table 1. There were no significant differences between the subjects with and without the polymorphism with regard to age, height, weight, BMI, waist circumference, systolic or diastolic blood pressure, fasting plasma glucose, triglyceride, or HDL cholesterol levels, PFC ratio (fat, carbohydrate), energy intake, physical activity, or smoker/non-smoker ratio. The total cholesterol values and PFC ratio (protein) of the subjects with the polymorphism were significantly lower than the subjects without the polymorphism (p=0.016 and 0.026, respectively).

**Table 1.** Clinical characteristics of the subjects, according to the ADRB3 polymorphism.

	All subjects	ADRB3 genotype		p value

		without polymorphism (Trp/Trp)	with polymorphism (Trp/Arg or Arg/Arg)	
n	295	198	97	
Age (years)*	46.1 ± 11.5	46.5 ± 11.3	45.3 ± 11.9	0.300
Height (cm)	168.6 ± 6.26	168.3 ± 6.19	169.1 ± 6.41	0.289
Weight (kg)	66.3 ± 10.4	66.3 ± 10.3	66.3 ± 10.6	0.999
Body mass index (kg/m^2^)	23.3 ± 3.28	23.4 ± 3.20	23.2 ± 3.47	0.633
Waist circumference (cm)	83.9 ± 9.30	84.1 ± 9.19	83.5 ± 9.57	0.636
Systolic blood pressure (mmHg)	134.4 ± 18.0	134.0 ± 18.5	135.2 ± 16.8	0.582
Diastolic blood pressure (mmHg)	81.7 ± 12.7	81.4 ± 13.1	82.3 ± 11.8	0.576
Fasting plasma glucose (mg/dL)^†^	100.2 ± 34.0	99.2 ± 31.8	102.5 ± 38.5	0.481
Total cholesterol (mg/dL)	206.9 ± 37.0	210.6 ± 35.8	199.3 ± 38.6	0.016
Triglyceride (mg/dL)^†^	131.9 ± 83.1	134.2 ± 86.1	127.1 ± 76.6	0.664
HDL cholesterol (mg/dL)	55.5 ± 14.0	55.5 ± 12.8	55.4 ± 16.4	0.976
Protein, fat, and carbohydrate ratio (%)				
Protein (%)	13.9 ± 2.39	14.1 ± 2.48	13.5 ± 2.14	0.026
Fat (%)	27.6 ± 5.36	27.7 ± 5.48	27.6 ± 5.13	0.890
Carbohydrate (%)	58.5 ± 6.88	58.2 ± 7.06	59.0 ± 6.51	0.379
Energy intake (kcal/day)	1855.3 ± 485.0	1857.3 ± 509.2	1851.3 ± 433.9	0.920
Physical activity (METs • minites)	2457.0 ± 710.9	2479.2 ± 763.8	2409.6 ± 583.6	0.442
Smoker/Non-smoker^‡^	175/119	112/85	63/34	0.184

Values are means±standard deviation. Subjects with polymorphism were compared with subjects without polymorphism.

Basically, we used Student’s *t*-test. As to variables with mark, we analyzed as follow.

* : Wilcoxon rank-sum test.

† : Variable was log transformed, and the Student’s *t*-test was used. Data is pre-log transformed.

‡ : *χ*^2^ test.

The subjects were classified into quartiles according to energy intake based on their replies to the FFQ. The mean waist circumference and BMI values in each quartile are shown in Table 2. There were no significant differences in waist circumference between the subjects in the 1st quartile and the subjects in the other quartiles. There was a significant difference in BMI between the subjects in the 1st quartile and in the 4th quartile (p=0.035), but not between the subjects in the 1st quartile and the subjects in the 2nd or the 3rd quartile. In addition, the trend test showed progressive increases in waist circumference and BMI in the quartiles that paralleled increased levels of energy intake (p=0.043 and 0.024, respectively).

**Table 2.** The mean waist circumference and body mass index values in each quartile, according to the energy intake.

	Energy intake				trend test

	1st quartile (-1515 kcal/day)	2nd quartile (1516-1795 kcal/day)	3rd quartile (1796-2131 kcal/day)	4th quartile (2132+ kcal/day)	
n	73	74	74	74	
Waist circumference (cm)	82.6±9.53	82.9±8.33	84.9±9.02	85.2±10.1	0.043
Body mass index (kg/m^2^)	22.6±3.28	23.2±3.24	23.6±3.33	23.8±3.24*	0.024

Values are means±standard deviation. Subjects in the 2nd, 3rd, and 4th quartile were compared with subjects in the 1st quartile. We used Student’s *t*-test and trend test.

* : P<0.05 for Student’s *t*-test.

We then divided each quartile into two groups according to presence of the polymorphism and calculated the ratio of the obese to the non-obese subjects in each group (Tables 3 and 4). When subjects with waist circumference 85+ cm^17^ were defined as obese (Table 3) in the 2nd quartile, the ratio of the group with the polymorphism was significantly lower than that of the group without the polymorphism (odds ratio [OR]=0.278, 95% CI=0.10-0.78), and in the 4th quartile, the ratio of the group with the polymorphism was significantly higher than that of the group without the polymorphism (OR=3.490, 95% CI=1.24-9.85).

**Table 3.** The ratio of the obese (waist circumference 85+ cm) to the non-obese (waist circumference less than 85 cm) by the ADRB3 polymorphism.

	ADRB3 genotype		both

	without polymorphism (Trp/Trp)	with polymorphism (Trp/Arg or Arg/Arg)	
1st quartile			
n (the obese/the non-obese)	24/26	8/15	32/41
the ratio of the obese to the non-obese	0.92	0.53	0.78
2nd quartile			
n (the obese/the non-obese)	24/21	7/22*	31/43
the ratio of the obese to the non-obese	1.14	0.32	0.72
3rd quartile			
n (the obese/the non-obese)	31/23	9/10	40/33
the ratio of the obese to the non-obese	1.35	0.90	1.21
4th quartile			
n (the obese/the non-obese)	21/27	19/7*	40/34
the ratio of the obese to the non-obese	0.78	2.71	1.18
all subjects			
n (the obese/the non-obese)	100/97	43/54	143/151
the ratio of the obese to the non-obese	1.03	0.80	0.95

The ratio of the obese to the non-obese in the group with the polymorphism was compared with that in the group without the polymorphism in each quartile. We used *χ*^2^ test.

* : P<0.05.

**Table 4.** The ratio of the obese (body mass index 25+ kg/m^2^) to the non-obese (body mass index less than 25 kg/m^2^) by the ADRB3 polymorphism.

	ADRB3 genotype		both

	without polymorphism (Trp/Trp)	with polymorphism (Trp/Arg or Arg/Arg)	
1st quartile			
n (the obese/the non-obese)	12/38	5/18	17/56
the ratio of the obese to the non-obese	0.32	0.28	0.30
2nd quartile			
n (the obese/the non-obese)	14/31	6/23	20/54
the ratio of the obese to the non-obese	0.45	0.26	0.37
3rd quartile			
n (the obese/the non-obese)	17/38	6/13	23/51
the ratio of the obese to the non-obese	0.45	0.46	0.45
4th quartile			
n (the obese/the non-obese)	16/32	11/15	27/47
the ratio of the obese to the non-obese	0.50	0.73	0.57
all subjects			
n (the obese/the non-obese)	59/139	28/69	87/208
the ratio of the obese to the non-obese	0.42	0.41	0.42

The ratio of the obese to the non-obese in the group with the polymorphism was compared with that in the group without the polymorphism in each quartile. We used *χ*^2^ test.

When subjects with BMI 25+ kg/m^2^ ^17^ were defined as obese, no significant difference between the ratio of obese to non-obese subjects in the group with the polymorphism and the group without the polymorphism was seen in any of the quartile (Table 4).

The results of the multiple logistic regression analysis on obesity (defined as waist circumference 85+ cm^17^) in the 4th quartile (total: 74, without polymorphism: 48, with polymorphism: 26) are shown in Table 5, in which the presence of *ADRB3* polymorphism, smoking, age and physical activity were independent variables. Only the presence of the polymorphism of *ADRB3* was associated with increased risk of obesity (adjusted OR=3.37, 95% CI=1.12-10.16). The results of the multiple logistic regression analyses on obesity defined as waist circumference 85+ cm^17^ and the same variables demonstrated that presence of the polymorphism of *ADRB3* was not associated with increased risk of obesity in the 1st, 2nd, and 3rd quartile.

**Table 5.** The multiple logistic regression analysis on obesity defined as waist circumference 85+ cm in the 4th quartile.

Explanatory variables	Adjusted odds ratio(95% confidence interval)
Presence of the polymorphism of *ADRB3*	3.37 (1.12-10.16)
Age (per year)	1.04 (1.00-1.08)
Smoking (smoker/non-smoker)	0.48 (0.17-1.37)
Physical activity (per 1000 METs•minutes)	0.76 (0.40-1.46)

Presence of the polymorphism of *ADRB3*, age, smoking, and physical activity were considered as variables.

## DISCUSSION

In this study, we classified the subjects into quartiles according to energy intake based on the replies to the FFQ. The values for energy intake based on the FFQ in this study were slightly lower than the National Nutrition Survey in 2002,^22^ which were based on diet records. The discrepancy is thought to be due to a failure to list all food items consumed by the subjects in the period due to the limitation on the number of food items listed in the FFQ. The FFQ has been reported to underestimate the absolute level of consumption of nutrients and food groups compared to diet records.^23^^,^^24^ However, it has been also reported that while the FFQ underestimates the absolute levels of consumption of nutrients and food groups, it is reasonably valid in ranking subjects and classifying them into quintiles according to the consumption of many nutrients and food groups.^23^ Furthermore, the percentages of complete agreement, adjacent agreement, and complete disagreement according to tertile classification of daily energy intake based on diet records and FFQ have been found to be 56%, 36%, and 7%, respectively.^24^ Thus, while energy intake based on the FFQ may only be a semi-quantitative index, it can be concluded to be an accurate means of classifying subjects into quartiles.

When the subjects were classified into two groups according to presence of the polymorphism, the total cholesterol values and PFC ratio (protein) of the subjects with the polymorphism were significantly lower than those of subjects without the polymorphism. Even if the function of the ADRB3 is considered,^7^ these results are of unexplained origin.

The trend test showed progressive increases in waist circumference and BMI in the quartiles that paralleled increased levels of energy intake (Table 2). Furthermore, when subjects with a waist circumference 85+ cm^17^ were defined as obese, the ratio in the group with the polymorphism was slightly but not significantly lower in the 1st and 3rd quartile, significantly lower in the 2nd quartile, and significantly higher in the 4th quartile (Table 3). These results indicate that increased energy intake may increase the risk of obesity without regard to the presence of the polymorphism of *ADRB3* and indicate that the combination of high energy intake and the presence of the polymorphism of *ADRB3* make this polymorphism a risk factor for obesity. Moreover, these results indicate that the combination of proper energy intake and the presence of the polymorphism of *ADRB3* may tend to reduce the risk of obesity. The hypothesis that the presence of the polymorphism of *ADRB3* alone does not affect the risk of obesity is supported by the finding that when the subjects were classified into two groups according to presence of the polymorphism, no significant difference in waist circumference or BMI was seen between the subjects with and without the polymorphism (84.1±9.19 vs 83.5±9.57, and 23.4±3.20 vs 23.2±3.47, respectively) (Table 1).

The results of the multiple logistic regression analysis showing that the presence of the polymorphism of *ADRB3* increases the risk of obesity in the 4th quartile alone (Table 5) also suggests interaction between high energy intake and the polymorphism of *ADRB3*.

A study comparing BMI, waist circumference, and waist-hip ratio (WHR) in regard to their respective associations with accumulation of abdominal visceral adipose tissue showed that the highest significant positive correlation was between waist circumference and abdominal visceral adipose tissue area measured by computed tomography in both men and women (r=0.77 and 0.87, respectively).^25^ When obesity was defined according to waist circumference not BMI in the present study, the interaction between the polymorphism of *ADRB3* and high energy intake was found to increase the risk of obesity. This finding indicates that the polymorphism of *ADRB3* is associated with visceral-fat obesity, which is supported by the finding that in humans *ADRB3* is predominantly expressed in visceral fat.^6^ The polymorphism of *ADRB3* has previously been reported to be associated with visceral-fat obesity,^13^^,^^26^ and the results of the present study do not contradict these reports.

Adipose tissue functions as a secretory tissue producing various adipocytokines including leptin, tumor necrosis factor-alpha (TNF-*α*), plasminogen activator inhibitor type I (PAI-1), and adiponectin.^27^^-^^30^ It is reported that adiponectin has anti-diabetic,^31^ anti-atherogenic,^32^ and anti-inflammatory biofunctions,^33^ and that the plasma levels of adiponectin decreased with the accumulation of visceral adipose tissue.^34^ It is also reported that intra-abdominal fat area was significantly associated with all of the metabolic syndrome criteria, including blood pressure, waist circumference, HDL cholesterol, triglyceride level, and fasting plasma glucose level,^35^ independent of insulin sensitivity and subcutaneous fat area. In addition intra-abdominal fat area was independently associated with development of metabolic syndrome (adjusted OR=2.43, 95% CI=1.33-4.47).^36^ Moreover, in the study based on 11 prospective European cohort studies involving 6156 men and 5356 women without diabetes, the overall hazard ratios for all-cause and cardiovascular disease mortality in subjects with the metabolic syndrome compared with those without the syndrome were 1.44 (95% CI=1.17-1.84) and 2.26 (95% CI=1.61-3.17) in men and 1.38 (95% CI=1.02-1.87) and 2.78 (95% CI=1.57-4.94) in women after adjustment for age, blood cholesterol levels, and smoking.^37^ Therefore, it is possible that prevention or treatment of visceral fat obesity, which is said to be associated with the polymorphism of *ADRB3*, could lead to reduced mortality associated with the metabolic syndrome.

The results of the present study demonstrated that the polymorphism of *ADRB3* alone does not increase the risk of obesity and that the environmental factor of high energy intake interacts with the polymorphism and leads to the significant increase in risk of obesity. This indicates the possibility that subjects with the Trp64Arg polymorphism can avoid the increase in the risk of obesity by proper energy intake control. Therefore, the Trp64Arg polymorphism may be a factor that we should take into consideration when we aim to tailor-made prevention or treatment of obesity. However, the findings that no significant difference in waist circumference or BMI was seen between the subjects with and without the polymorphism indicate that the effect of the polymorphism in the development of obesity is rather small and that it may be difficult to carry out tailor-made prevention or treatment of obesity based on genotyping of the Trp64Arg polymorphism of *ADRB3* alone. Further studies investigating the interaction between the Trp64Arg polymorphism of *ADRB3* and other polymorphisms involved in the development of obesity, will be needed.
